# Correlation Between Emotional Intelligence and Readiness for Self-Directed Learning in Medical Undergraduate Students

**DOI:** 10.7759/cureus.62716

**Published:** 2024-06-19

**Authors:** Vijaya Lakshmi, John A Lyngdoh

**Affiliations:** 1 Physiology, North Eastern Indira Gandhi Regional Institute of Health and Medical Sciences, Shillong, IND

**Keywords:** self-directed learning readiness, life-long learner, self-directed learning, cbme curriculum, emotion, emotional intelligence

## Abstract

Background: The success of self-directed learning depends mainly on the readiness of students to adapt it to their learning domain. Medical students must meet certain criteria to become self-directed learners, which are also significant components of emotional intelligence (EI). Clarification is required on whether the students are ready for self-directed learning according to their level of EI as soon as they enter the medical institute.

Materials and methods: The survey was conducted on first-year MBBS students, between 18 and 21 years of age. Demographic data of the participants was collected. EI was assessed by using the Schutte Self-Report Emotional Intelligence Test (SSEIT). Fisher’s 40-item self-directed learning readiness (SDLR) scale was used to assess the readiness for self-directed learning. Pearson’s correlation and regression analysis was carried out to assess the relationship between the two.

Result: Approximately 71% of students had average EI, whereas only 5% had high EI. However, 63% of students were found to have low SDLR, while just 37% of participants had high SDLR. EI and SDLR both were found to be higher in males. Pearson’s correlation “r” between the two parameters shows a strong positive correlation with statistical significance.

Conclusion: Certain training modules need to be incorporated into the medical education program to improve the EI of medical undergraduate students. Such a module might help in improving the readiness for self-directed learning and prepare the medical undergraduates as active lifelong learners, which is the prime goal for an Indian Medical Graduate according to the new Competency-Based Medical Education (CBME) curriculum.

## Introduction

Self-directed learning (SDL) is a new concept of teaching-learning methodology that was introduced by the National Medical Commission (NMC) in the Competency-Based Medical Education (CBME) Curriculum in 2019 [1]. SDL concept for medical undergraduate students is an active learning method that serves to develop their independent learning skills and increase their sense of responsibility. The new CBME curriculum is said to be learner-centric as compared to the traditional curriculum which was teacher-centric. Therefore, it requires the active involvement of the learner to become a lifelong learner (LLL), which is the goal for an Indian Medical Graduate as enshrined in the new curriculum.

SDL is defined as “a process by which an individual takes the initiative to identify his/her learning needs, frame learning goals, implement appropriate learning strategies, and evaluate his/her learning outcome” [2]. Task-specific goals must be kept in mind for the successful completion of SDL by students. They need to plan and organize their learning skills. SDL plays an important role in improving confidence, self-determination, communication skills, and learning on their own by trial and error [3]. Various modalities of SDL include problem-based learning, flipped classrooms, simulation laboratories, online resources, etc. The designing of SDL sessions and their successful implementation is a great challenge for the facilitators. Barriers to this learning modality may include student’s lack of awareness, interest, motivation, type of prior schooling, access to the internet, self-confidence, curiosity, decision-making ability, etc. Assessment of SDL sessions also forms a big challenge for the facilitators.

The success of SDL depends mainly on the readiness of students to adapt the same in their learning domain. SDL readiness (SDLR) is the degree to which a student possesses the above qualities. Thus, it can be mentioned that the measurement of SDLR is essential for the education plan of medical undergraduates [4].

SDLR is thus the measure of the level an individual possesses attitudes, abilities, and personality characteristics necessary for SDL [5]. SDLR score represents the overall measure of a student’s inclination toward SDL while the scores in various domains relate to the student’s specific strengths and weaknesses.

Researchers have often mentioned that emotion is the foundation of learning [6] and thus has an important impact on the learning process. Emotion, however, is said to be a double-edged sword, as it not only serves as a motivator to enhance learning but also prevents one from learning effectively. Learners often differ in how they manage their emotions, which can either motivate them to learn or discourage their learning process. Therefore, learning outcomes are affected severely by their Emotional Intelligence (EI) [7]. EI is the ability to keep track of self as well as others’ feelings or emotions and to use them judiciously for one’s thinking and actions [8].

Bar-On mentioned that EI is composed of self-perception, self-expression, interpersonal skills, decision-making skills, and stress management [9] EI is said to play an important role not only in improving communication skills or job satisfaction but also in influencing academic and clinical performance of a medical graduate, thus helping in building a good doctor-patient relationship [10].

Zhoc et al. studied the role of EI on SDL and found that students who are more emotionally intelligent are more self-directed, thus attaining higher academic achievement [11]. There are certain criteria for medical students to become self-directed learners which also form important components of EI. These criteria include critical thinking, reflection, critical appraisal, information management, teamwork, self - as well as peer evaluation, and identification of their learning gaps.

Arora et al. found that higher EI plays an important role in improving doctor-patient interactions and teamwork skills [12]. Therefore, we need to clarify whether the students are ready for SDL according to their level of EI as soon as they enter the Medical Institute. The effects of EI on SDL need to be assessed and how EI and SDL can contribute to key learning outcomes in medical education in India.

## Materials and methods

Study design

This was a descriptive, cross-sectional, questionnaire-based study that applied a convenient sampling method for recruitment.

Inclusion criteria

First-year MBBS students at our medical school who expressed willingness to take part in the study.

Exclusion criteria

Students who declined to be included in the study and those who provided incomplete details.

Instrument

The survey was conducted among first-year MBBS students between 18 and 21 years of age. Students were assured that their participation in the study would be anonymous and confidential. The survey was conducted after obtaining approval from the Institutional Ethics Committee for Human Studies. Students were informed that participation in the study was voluntary, and they could choose to exit from the study whenever they wished to. Written instructions were given. Students were requested to be honest in their responses. Demographic data of the participants like age and gender were collected.

EI was assessed by using the Schutte Self-Report Emotional Intelligence Test (SSEIT). The students were instructed to carefully go through the statements and score them on the Likert scale from 1 to 5, which has 1=strongly disagree to 5=strongly agree. Three of the 33 statements, viz., 5, 28, and 33 were reverse scored. A total score less than 111 was considered low and a score above 137 was a high EI. A score between 111 and 137 was considered an average EI score [13]. The subscales of EI including all 33 items are as follows: Perception of Emotion, Managing Own Emotions, Managing Others’ Emotions, and Utilization of Emotion [14].

The SDLR Scale utilized was designed by Fisher et al. in 2001. It is a validated tool and is used to measure the degree to which a student possesses the attitude and ability necessary for SDL. Fisher’s SDLRS has 40 items grouped into three sub-scales, i.e., “Self-management,” “Desire for learning,” and “Self-control,” and are rated on a 5-point Likert scale [15].

Statistical analysis

All the data including demographic details, SSEIT score, and Fisher’s SDLR scale were collected and entered on a Microsoft Excel sheet and thereby analyzed. Pearson’s correlation analysis was done to assess the correlation between the EI Score and the SDLR Score. Regression analysis was done to evaluate the statistical significance of the collected data. P-values < 0.05 were considered significant.

## Results

A total of 110 students participated in the study, out of which three submitted incomplete questionnaires. Forty-three females and 64 male students with a mean age of 19.67± 1.15 years participated in the current study.

Table 1 shows the lowest and highest values of EI in both male and female students as well as the mean values with standard deviation (Mean ± SD). The highest EI value was found to be the same for both male and female students, though the mean EI of male students was more than their female counterparts in the current study.

**Table 1:** Gender differences in the distribution of emotional intelligence EI - emotional intelligence

S No	Gender	Lowest	Highest	Mean±SD
1	Male	97	157	120.65 ± 12.18
2	Female	99	157	117.11 ± 11.5
3	Overall EI	97	157	119.19 ± 11.97

**Figure 1:**
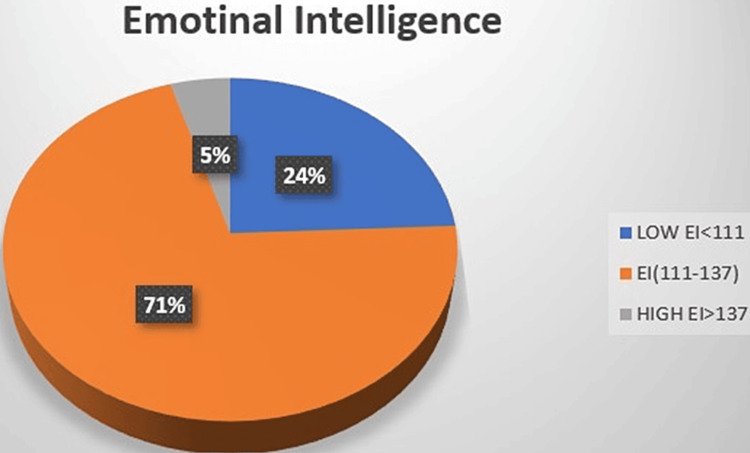
Distribution of score for emotional intelligence EI - emotional intelligence

Figure 1 shows the distribution of EI scores. Around 71% of participants had an average EI score, 24% with low EI score and just 5% had a high EI score. Hence, it is imperative to develop modalities for improving the EI of these students as 95% of them have average or low EI.

Table 2 represents the distribution of a domain-wise range of EI scores and the mean and standard deviation for each domain. The score for Perception of Emotion being the lowest emphasizes the need to train students to be aware of their emotions and manage them successfully.

**Table 2:** Domain-wise score for emotional intelligence

S No	Domain	Score Range	Mean ± SD
1	Perception of Emotion	10 - 55	34.74 ± 7.8
2	Managing own Emotions	9 - 45	33.44 ± 6.96
3	Managing others' Emotions	8 - 40	28.48 ± 6.08
4	Utilization of Emotion	6 - 30	22.54 ± 4.41

Table 3 shows the gender-wise distribution of readiness for SDL with male students having higher readiness scores as compared to the female students in the current study.

**Table 3:** Gender differences in self-directed learning readiness (SDLR)

S No	Gender	Mean ± SD
1	Overall	145.82 ± 12.31
2	Male	148.61 ± 11.72
3	Female	141.61 ± 12.13

**Figure 2:**
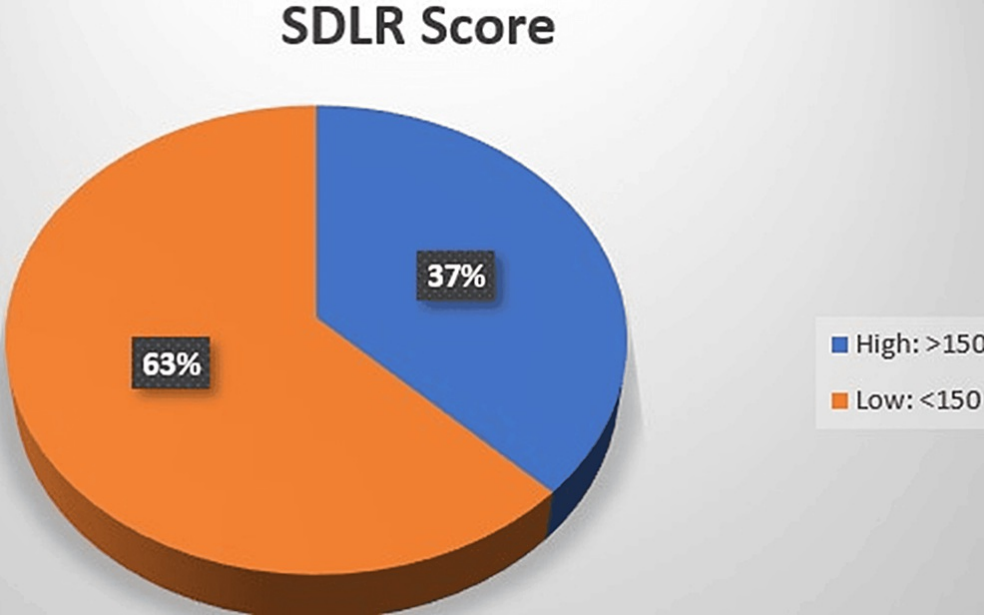
Score of readiness for self-directed learning

Figure 2 shows that 63% of students had low SDLR scores. Only 37% of students had high readiness for SDL, i.e., they could independently learn through proper planning, management strategy, etc. Students with low SDLR need to develop skills for enhancing self-confidence as well as time management.

Table 4 represents the mean and standard deviation of various domains of SDLR, i.e., self-management, desire for learning, and self-control. Self-control was the highest whereas scores for self-management and desire for learning were less but nearly similar. Self-management skill needs to be imparted to the students as well as desire for learning needs to be aroused among them.

**Table 4:** Scores for various dimensions of self-directed learning readiness SDLR - self-directed learning readiness

S No	Domain	Range	Mean ± SD
1	Self-Management	13 to 65	45.01 ± 4.59
2	Desire for Learning	12 to 60	45.26 ± 4.15
3	Self-Control	15 to 75	55.55 ± 5.29
4	Overall SDLR	40 to 200	145.82 ± 12.31

Table 5 represents the conduction of correlation analysis to determine the relationship between the SDLR & EI Score based on the research question “Is there any relationship between SDLR and EI score of first-year medical undergraduate students”. Pearson’s correlation “r” between the two parameters is 0.787 which represents a strong positive correlation between SDLR and EI score.

**Table 5:** Pearson’s correlation analysis of SDLR and EI score SDLR - self-directed learning readiness, EI - emotional intelligence

	EI Score	SDLR
EI Score	1	0.787741
SDLR	0.787741	1

Table 6 represents the regression analysis of the SDLR and EI score to assess whether the relationship is significant or not. The relationship between the two was found to be statistically significant as p-value < 0.05.

**Table 6:** Regression analysis of SDLR and EI score SDLR - self-directed learning readiness, EI - emotional intelligence

Regression statistics						
	Coefficients	Standard Error	t-Stat	P-value	Lower 95%	Upper 95%
Intercept	31.2120297	8.90250333	3.5059835	0.00067	13.56001016	48.8640493
EI Score	0.86619394	0.06649925	13.025619	1.17E-23	0.734338208	0.99804967

## Discussion

In the current study, the mean value of EI of male medical undergraduate students was found to be comparatively higher than female students. Though some studies have reported females with higher EI, the current study is consistent with the previous study where males were found to have higher EI [12]. It was seen that just 5% of students had high EI and 24% had low EI. Most of the students, i.e., 71% had average EI scoring between 111 and 137.

As 71% of the study population had average EI, it is imperative that we need a mechanism to train such students to improve their EI. This can be achieved by training the medical students by organizing workshops on EI skills, personal and interpersonal skills, and developing courses on handling emotions. It is said that higher EI is associated with good academic performance, better patient-physician relationships, increased empathy, improved teamwork and communication abilities, stress management, and leadership qualities [12]. The new CBME Curriculum by NMC has introduced a Foundation course, attitude, ethics, and communication (AETCOM) skill module, Family adoption program, etc. These steps will also help in improving the EI of medical undergraduates and prepare them as more competent medical professionals.

In the current study, the highest score is seen for utilization of emotion and the lowest for perception of emotion when we calculated the domain-wise score of EI. In previous studies, it has been mentioned that someone with a good EI is more aware of his own emotions and thus capable of facing hardships courageously which could have otherwise triggered stress, anxiety, or even depression.

SDL is a process in which an individual takes the initiative to fulfill the learning needs, identifies the resources, formulates goals, and develops appropriate strategies for achieving better learning outcomes [16,17]. SDL is very challenging and demanding as young students not only have to manage their overall learning activities but also monitor their own performance [4,18]. Thus, it is often challenging even for the brightest, motivated, and most responsible student.

It has been mentioned that a high level of SDLR shows that the student is ready to learn independently. Such students possess better behavior, ability, character, and management strategies and can practice independent learning as well [19]. In the current study, readiness for SDL is seen to be more in the male participants as compared to the female participants. Though the overall score of the participants is less than 150, which means that these participants have less Readiness for SDL. The reason can be that they have just entered the medical college after several years of schooling, i.e., pedagogical teaching. Therefore, these students need to be trained to be self-sufficient in their learning needs and thereafter SDL sessions can be started. This will help them become a better LLL and face the challenges in their career confidently.

In a previous study by Nyambe et al. in 2016, it was reported that students with a moderate level of SDLR were seen to be more dependent on the faculty for their learning process or tend to study only with the approaching exam [20]. They were unable to manage themselves well in terms of time management or self-control from any kind of distraction. In such a situation, the concerned teacher or facilitator needs to motivate the students as motivation helps the students to participate and even sustain the will to work hard to achieve their goals [21].

While calculating the various domains of SDLR, it was seen that the participants scored more for self-control as compared to self-management and desire for learning in this study. This can be inferred as they can well control the difficult situations in their life but the desire for learning themselves needs to be aroused. This can happen only with well-trained faculty who can guide them in managing their study pattern and thereafter make them LLL, which is an important requisite for a good medical professional.

Pearson’s correlation “r” shows a strong positive correlation between SDLR Score and EI. This relation is statistically significant as p-value is < 0.05. Thus, in the current study, it can be emphasized that most of the students are with average EI and their Readiness for SDL is accordingly low. The Facilitators need to help them improve their learning skill and guide them in making critical decisions related to not only their studies but also in facing difficult situations in life and thus prepare them as a successful medical professional.

 A successful medical student needs to acquire several learning skills such as confidence, autonomy, motivation, and preparation for lifelong learning [22]. SDL forms an essential skill for medical students which helps in developing higher order thinking as well as problem-solving capacity.

This study emphasizes that a good Emotional state is the prime requisite for improving the Readiness for SDL. It can thus maximize the cognitive abilities of medical undergraduates as well as boost their levels of SDLR and prepare them as LLL in the future.

Limitation

The study population in the current study consists of first-year students from a single institution, i.e., a small sample size. Hence, its findings cannot be generalized. The data obtained in this study are based on the self-report by the students which may not reflect the true values and may be subject to variation. The longitudinal study might be required to assess if there is any change in their EI and SDLR as they proceed from the first year to the final year of their graduation.

## Conclusions

Several studies have been carried out to assess either the EI or the readiness for SDL of medical undergraduate students. No literature has been found yet to correlate the two in Indian Medical Graduates. In the current study, it was found that 71% of students have average EI and 63% have low SDLR and there is a strong positive correlation with statistical significance between the two. Therefore, certain training modules can be incorporated into the medical education program to improve EI in these students. Such training will also help in improving their readiness for SDL and help them become good LLLs. Once trained to handle emotions of self and others, medical students can achieve not only academically but also professionally.

## Figures and Tables

**Figure 3:**
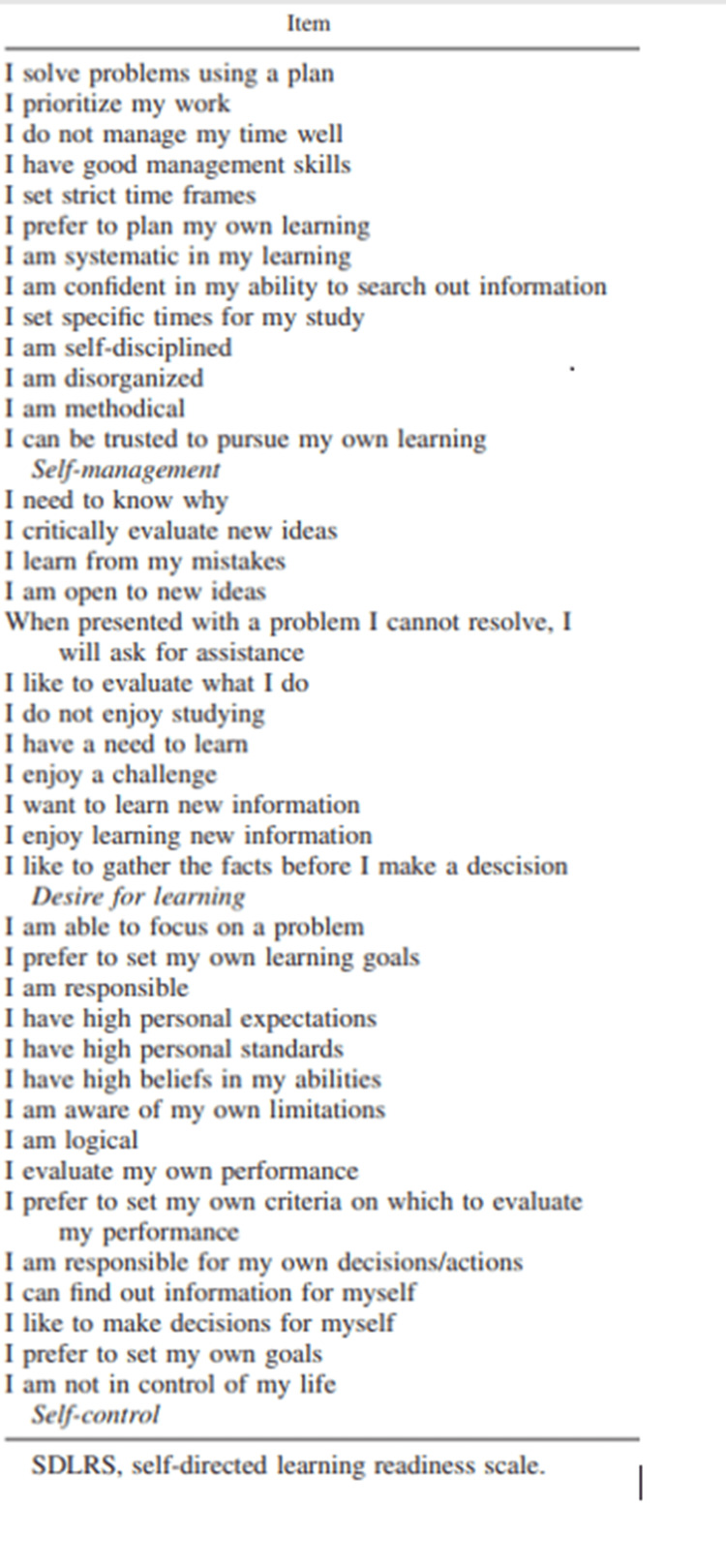
Fisher's SDLR scale (Questionnaire)

**Figure 4:**
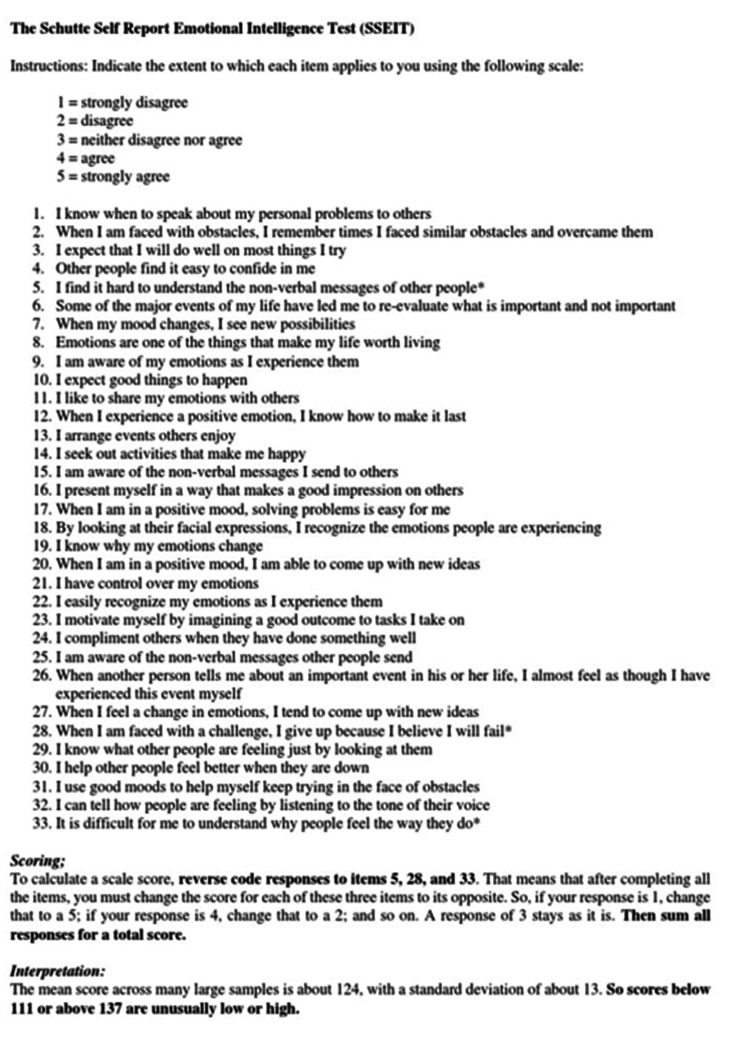
Schutte Self-Report Emotional Intelligence Test (SSEIT) Questionnaire

## References

[1] Medical Council of India (MCI) (2018). Competency Based Undergraduate Curriculum for the Indian Medical Graduate. Vol. 1. MCI; Medical Council of India (MCI.

[2] Knowles M (1975). New York, USA: Association Press.

[3] Kindy S, Kindy F, Kindy AAI (2018). The advantages and disadvantages of self-directed learning: a survey - study of Saudi medical students. MedEdPublish.

[4] Premkumar K, Vinod E, Sathishkumar S, Pulimood AB, Umaefulam V, Prasanna Samuel P, John TA (2018). Self-directed learning readiness of Indian medical students: a mixed method study. BMC Med Educ.

[5] Wiley K (1983). Effects of a self-directed learning project and preference for structure on self-directed learning readiness. Nursing Research.

[6] Zull JE (2006). Key aspects of how the brain learns. Wiley Online Lib.

[7] King RB, Chen J (2019). Emotions in education: Asian insights on the role of emotions in learning and teaching.. Asia Pacific Educ Res.

[8] Mayer JD, Salovey P, Caruso DR (2008). Emotional intelligence: new ability or eclectic traits?. Am Psychol.

[9] Bar-On R (2006). The Bar-On model of emotional-social intelligence (ESI). Psicothema.

[10] Weng HC, Hung CM, Liu YT, Cheng YJ, Yen CY, Chang CC, Huang CK (2011). Associations between emotional intelligence and doctor burnout, job satisfaction and patient satisfaction. Med Educ.

[11] Zhoc KC, Chung TS, King RB (2018). Emotional intelligence (EI) and self-directed learning: examining their relation and contribution to better student learning outcomes in higher education. British Educ Res J.

[12] Arora S, Ashrafian H, Davis R, Athanasiou T, Darzi A, Sevdalis N (2010). Emotional intelligence in medicine: a systematic review through the context of the ACGME competencies. Med Educ.

[13] Schutte NS, Malouff JM, Hall LE, Haggerty DJ, Cooper JT, Golden CJ, Dornheim L (1998). Development and validation of a measure of emotional intelligence. Personality Individual Diff.

[14] Ciarrochi J, Chan AYC, Bajgar J (2001). Measuring emotional intelligence in adolescents. Personality Individual Diff.

[15] Fisher M, King J, Tague G (2001). Development of a self-directed learning readiness scale for nursing education. Nurse Educ Today.

[16] Murad M, Coto-Yglesias F, Varkey P, Prokop L, Murad A (2010). The effectiveness of self-directed learning in health professions education, a systematic review. Med Educ.

[17] Williams B, Boyle M, Winship C, Brightwell R, Devenish S, Munro G (2013). Examination of self-directed learning readiness of paramedic undergraduates: a multi-institutional study. J Nurs Educ Practice.

[18] Leatemia LD, Susilo AP, van Berkel H (2016). Self-directed learning readiness of Asian students: students perspective on a hybrid problem based learning curriculum. Int J Med Educ.

[19] Fisher MJ, King J (2010). The self-directed learning readiness scale for nursing education revisited: a confirmatory factor analysis. Nurse Educ Today.

[20] Nyambe H, Mardiwiyoto H, Rahayu GR (2016). Factors that influence self directed learning readiness in first, second- and third-year students at the Faculty of Medicine, Hasanuddin University in PBL. Indonesian J Med Educ.

[21] Corno L (1992). Encouraging students to take responsibility for learning and performance. Elementary School J.

[22] Jacobs JL, Samarasekera DD, Shen L, Rajendran K, Hooi SC (2014). Encouraging an environment to nurture lifelong learning: an Asian experience. Med Teach.

